# Absolute quantitative metagenomic analysis reveals unique gut bacteria underlying berberine and metformin’s anti-metabolic disorders effects

**DOI:** 10.1128/spectrum.00084-25

**Published:** 2025-07-31

**Authors:** Jiaguo Zhan, Bin Cheng, Kangmiao Guo, Xueqing Tao, Xinyan Cai, Zhuoran Li, Zhenhao Tang, Jingke Zhan, Chongming Wu

**Affiliations:** 1 School of Chinese Materia Medica, Tianjin University of Traditional Chinese Medicine 58301, Tianjin, China; 2 School of Nursing, Tianjin University of Traditional Chinese Medicine 58301, Tianjin, China; 3 State Key Laboratory of Chinese Medicine Modernization, Tianjin, China; 4 Tianjin Key Laboratory of Therapeutic Substance of Traditional Chinese Medicine, Tianjin, China; National Center for Genetic Engineering and Biotechnology, Khlong Luang, Pathum Thani, Thailand

**Keywords:** diet-induced metabolic disorder, berberine, metformin, absolute quantitative sequencing, relative quantitative sequencing

## Abstract

This study aimed to evaluate the potential functional bacteria in the efficacy of two drugs in ameliorating diet-induced metabolic disorder model using absolute and relative quantification methods and to evaluate the benefit of absolute quantification compared with relative quantification. The gut microbiota is implicated in the pathogenesis of various chronic diseases, including diet-induced metabolic disorder model. Berberine (BBR) and metformin (MET) are commonly used in the clinical management of metabolic disorder, yet their effects on gut microbiota regulation differ. This study employs both relative and absolute quantitative methods to assess the differential impacts of these drugs on the modulation of gut microbiota in metabolic disorder mice. Both BBR and MET effectively ameliorate the condition of metabolic disorder. While some relative quantitative sequencing results contradicted the absolute sequencing data, the latter was more consistent with the actual microbial community composition. Absolute quantitative sequencing provides a more accurate reflection of the drug’s effects. Notably, both absolute and relative quantitative sequencing demonstrated an upregulation of *Akkermansia*.

**IMPORTANCE:**

Our study underscores the importance of absolute quantitative analysis in accurately representing the true microbial counts in a sample and evaluating the modulatory effects of drugs on the microbiome, which plays a vital role in the study of the microbiome.

## INTRODUCTION

The gut microbiota plays a critical role in the onset and progression of various diseases, as well as in the effectiveness of therapeutic interventions (1, 2). While several quantitative methods are currently available to detect microbiome variations linked to disease, their capacity to fully elucidate the complex interactions between the microbiota and host health remains limited (3, 4). Current gut microbiota sequencing studies predominantly employ relative quantitative approaches, which analyze differences in the V1–V9 hypervariable regions of the 16S rRNA gene across multiple biological samples (5). This method generates compositional data by normalizing the sum of all detected features to unity, thereby determining the relative proportions of target genes or taxa without requiring absolute copy numbers. It is extensively applied in pharmacological modulation of gut microbiota, discovery of disease-associated microbial signatures, and integration with proteomic biomarker identification (6, 7). Absolute quantitative sequencing necessitates precise measurement of microbial DNA concentration and copy numbers, providing taxon-specific absolute counts rather than proportional data, thereby achieving enhanced sensitivity in detecting low-abundance species (8). Current methodologies primarily involve spike-in internal standards (e.g., exogenous microbial DNA with predefined quantities) (9), flow cytometry for bacterial DNA quantification via fluorescent labeling (10), and quantitative PCR (qPCR) targeting species-specific genetic markers (11). This approach is now commonly employed in studies exploring host-microbe metabolic interactions, environmental microbiome profiling (including bacterial, fungal, and eukaryotic community abundances) (12, 13).

Analytical comparisons based on relative microbiome data fail to reveal the extent or direction of changes in taxon abundance or metabolic potential. Additionally, they may hinder the ability to correlate microbiome characteristics with quantitative data, especially when microbial loads differ significantly between samples (e.g., physiological parameters or metabolite concentrations) (14, 15). Furthermore, relative abundance methods overlook fluctuations in overall microbiota abundance, potentially missing key markers of disease-related shifts in ecosystem structure (16). Moreover, several studies have demonstrated that changes in the relative abundance of microbiota do not necessarily reflect absolute changes in abundance and that relative and absolute abundance measures are often poorly correlated (17). There is growing evidence that measuring the total number and concentration of microorganisms in the gut, known as “absolute abundance,” provides more comprehensive insights than “relative abundance.” This approach can correct potential inaccuracies that arise from analyses based solely on relative abundance data (11). Furthermore, under the influence of various drugs, the absolute number of bacteria may undergo significant changes, whereas relative quantification fails to accurately capture these differences in absolute bacterial counts. Overall, absolute quantification of microorganisms provides a more robust and precise understanding compared to relative quantitative methods.

In recent years, the incidence of metabolic syndrome, including type 2 diabetes (T2D) and hyperlipidemia, has been steadily increasing. The complications associated with these conditions significantly affect human quality of life and overall health (18, 19), whereas the microbiome is implicated in the pathology of many chronic diseases, including metabolic disorders (20, 21). A substantial body of evidence suggests that microbiota, such as probiotics and prebiotics, influence glucose metabolism in both preclinical animal models of metabolic disorders and healthy animals (22, 23).

Studies have shown that drugs such as BBR and MET can modulate the gut microbiota by upregulating the relative abundance of beneficial genera, including *Lactobacillaceae*, *Spirochaetaceae*, *Bacteroides*, *Bifidobacterium*, *Lactobacillus*, and *Akkermansia*, while simultaneously decreasing the abundance of conditional pathogens (*Verrucomicrobiaceae* and *Enterobacteriaceae*) (24, 25); promote the increase of beneficial metabolites, including short-chain fatty acids and enhance intestinal GLP-1 secretion (24, 26); and further impact bile acid and amino acid metabolism, as well as intestinal immunity to ameliorate diabetes and hyperlipidemia (27). Berberine is well known for its antimicrobial properties (28), while metformin effectively supports the proliferation of short-chain fatty acid-producing bacteria such as *Akkermansia muciniphila* and *Bifidobacterium bifidum* in the gastrointestinal tract (29). Therefore, comparing the effects of BBR and MET on the gut microbiota is valuable for distinguishing the differences between relative and absolute quantitative methods in microbiological analysis.

In this study, we aimed to elucidate the distinctions between relative quantitative sequencing and absolute quantitative sequencing by investigating the regulatory effects of MET and BBR on the gut microbiota in metabolic disorders. Initially, we reaffirmed the beneficial effects of MET and BBR on high-fat diet-induced metabolic disorders. Subsequently, we assessed the differing regulatory impacts of these two compounds on the microbiota using both relative and absolute quantitative sequencing methods, and we compared our sequencing results. Our research underscores the importance of absolute quantitative analysis in accurately representing the true microbial counts in samples and assessing the regulatory effects of drugs on the microbiome, while also holding significant implications for understanding gut microbiota and developing probiotic approaches targeting metabolic disorders.

## MATERIALS AND METHODS

### Animal research

Male ICR mice, acquired from StemBioSys Biotechnology Co., Ltd. (Beijing, China), were housed under conditions consistent with previous studies (12). All experimental procedures were adhered to the ethical guidelines stipulated by the Academic Committee on Animal Experiment Ethics at Tianjin University of Traditional Chinese Medicine (Approval No.: TCM-LAEC2023231z1632). Forty-eight SPF grade ICR mice were randomly divided into four groups as follows: Control group, Model group, Berberine group (BBR, 0.1 g/kg), and Metformin group (MET, 0.3 g/kg).

A model of metabolic disorder was established in mice through a high-sugar and high-fat diet for 6 weeks (67% standard chow, 10% lard, 20% sucrose, 2.5% cholesterol, and 0.5% sodium cholate). The treatment commenced 1 week after the initiation of the high-fat diet. The control and model groups received normal saline via gavage, while the MET group was administered metformin at a dosage of 0.3 g/kg, and the BBR group received berberine at a dosage of 0.1 g/kg. Blood samples were collected from the tail weekly to monitor changes in fasting blood glucose levels in the mice. A successful model was defined as having fasting blood glucose levels exceeding 7.1 mmol/L, marking the experimental endpoint. Upon completion of the experimental protocol, mice were euthanized under anesthesia induced by 2% sodium pentobarbital. Terminal blood samples were collected via retro-orbital puncture. Liver tissues were immediately excised, rinsed with ice-cold PBS (Solarbio, China), and processed for either histopathological fixation (4% paraformaldehyde, Solarbio) or snap-freezing at −80°C for further analyses. Concurrently, cecal tissues along with luminal contents were harvested and flash-frozen at −80°C to ensure integrity for subsequent full-length 16S rRNA gene sequencing.

### Biochemical index detection

Mouse liver samples were processed according to the instructions of the biochemical kit of Nanjing Jiancheng Bioengineering Institute, and the levels of blood and liver biochemical indicators, including blood glucose (FBG, A154-1-1), total cholesterol (TC, A111-1-1), triglycerides (TG, A110-1-1), liver glycogen (A043-1-1), serum insulin (H203-1-1), high-density lipoprotein cholesterol (HDL-C), and low-density lipoprotein cholesterol (LDL-C, A113-1-1), were detected.

### Histological examination

The mice were euthanized. Harvest the liver and wash it in cold PBS before draining it with absorbent kitchen paper. Liver tissue samples fixed in 4% paraformaldehyde were trimmed, dehydrated, and embedded in paraffin wax, followed by sectioning using a cryotome, and the 5-μm-thick slices were stained with hematoxylin and eosin (H&E) for subsequent. The slides were scanned with a Lycra biopsy scanner.

### Full-length 16 S rRNA gene sequencing

Fecal DNA extraction was conducted following the methodology established in previous studies (1). The V1–V9 hypervariable regions of the 16S rRNA gene were amplified using the primer pair 27F (5′-AGRGTTYGATYMTGGCTCAG-3′) and 1492R (5′-RGYTACCTTGTTACGACTT-3′). Following PCR, amplicons were purified from 2% agarose gels, and SMRTbell libraries were constructed via blunt-end ligation (Pacific Biosciences protocol). Sequencing was performed on the PacBio Sequel II platform (Genesky Biotechnologies Inc., Shanghai, 201315, China). Raw FASTA files were subjected to quality filtering and sequence alignment, followed by amplicon sequence variant (ASV) clustering at 97% similarity. Downstream analyses—including relative abundance, alpha diversity (e.g., Shannon index), and beta diversity (e.g., PCoA)—were computed in R (v4.2.3).

### Accurate 16S absolute quantification sequencing

Accu16STM (Accurate 16S absolute quantification sequencing) was performed by Genesky Biotechnologies Inc., Shanghai, 201315 (China). Briefly, total genomic DNA was extracted using the FastDNA SPIN Kit for Soil (MP Biomedicals, Santa Ana, CA) according to the manufacturer’s instructions. The integrity of genomic DNA was detected through agarose gel electrophoresis, and the concentration and purity of genomic DNA were detected through the Nanodrop 2000 and Qubit3.0 Spectrophotometer. Multiple spike-ins with identical conserved regions to natural 16S rRNA genes and variable regions replaced by random sequence with ~40% GC content were artificially synthesized. Then, an appropriate proportion of spike-ins mixture with known gradient copy numbers was added to the sample DNA. The V3–V4 hypervariable regions of the 16S rRNA gene and spike-ins were amplified with the primers 341F (5′-CCTACGGGNGGCWGCAG-3′) and 805R (5′-GACTACHVGGGTATCTAATCC-3′) and then sequenced using Illumina NovaSeq 6000 sequencer.

### Illumina read data processing and analysis

The raw read sequences were processed in QIIME2 (30). The adaptor and primer sequences were trimmed using the cutadapt plugin. DADA2 plugin was used for quality control and to identify ASVs (31). Taxonomic assignments of ASV representative sequences were performed with confidence threshold 0.8 by a pre-trained Naive Bayes classifier which was trained on the Greengenes (version 13.8). Then, the spike-in sequences were identified and reads were counted. Standard curve for each sample was generated based on the read-counts vs spike-in copy number, and the absolute copy number of each ASV in each sample was calculated by using the read-counts of the corresponding ASV. Since the spike-in sequence is not a component of the sample flora, the spike-in sequence needs to be removed in the subsequent analysis (32).

### Statistical analysis

All statistical evaluations were orchestrated via GraphPad Prism 8 (GraphPad, San Diego, CA). The data were parsed using one-way analysis of variance (ANOVA) and were represented as mean ± SEM; the post-hoc analysis following one-way ANOVA was performed using Tukey’s Honestly Significant Difference (HSD) test. Results were deemed statistically significant when *P* value was under 0.05.

## RESULTS

### MET and BBR ameliorate glucose and lipid metabolism in metabolic disorder mice

**Fig 1:**
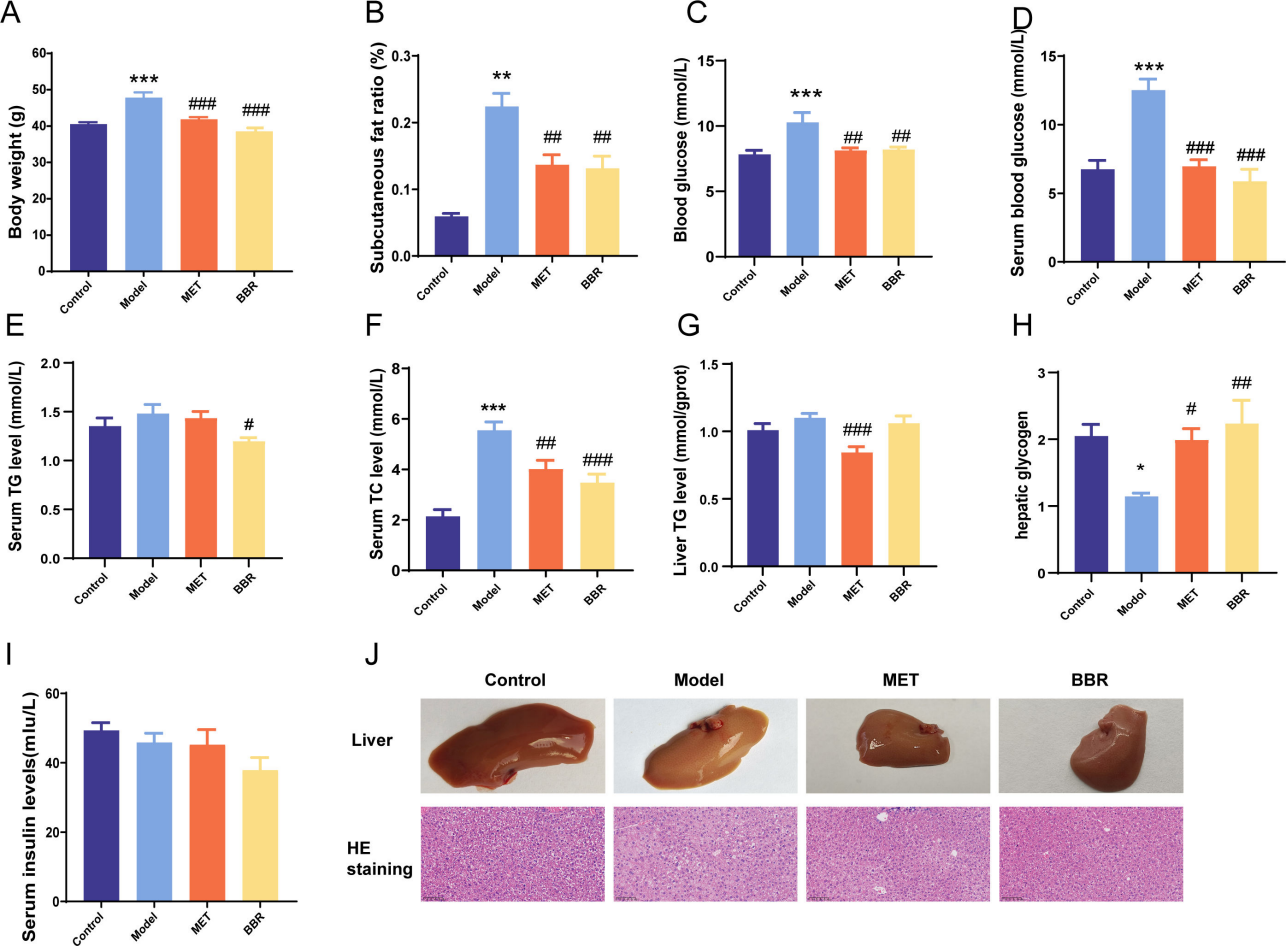
BBR and MET relieve metabolic disorders. (**A**) Body weight on 9th day. (**B**) Subcutaneous fat ratio. (**C**) Fasting blood glucose. (**D**) Serum blood glucose levels in different groups. (**E**) Serum TG levels in different groups. (**F**) Serum TC levels in different groups. (**G**) TG levels in livers of different groups. (**H**) TC levels in livers of different groups. (**I**) Liver glycogen levels in different groups. (**J**) Liver morphology and HE staining in different groups. *N* = 8 for each group. *P < 0.05, **P < 0.01, ***P < 0.001.

To further investigate the effects of BBR and MET on glucose and lipid metabolism in metabolic disorder mice, we treated mice with both drugs for 7 weeks and assessed various serum biochemical markers. Both BBR and MET administration significantly reduced body weight, fasting blood glucose levels, and abdominal fat ratio compared to the model group (Fig. 1A through C). Furthermore, treatment with BBR and MET notably decreased serum triglycerides (TG), total cholesterol (TC), and low-density lipoprotein (LDL) levels, while increasing high-density lipoprotein (HDL) levels. Additionally, hepatic TG levels were reduced, and hepatic glycogen content was elevated (Fig. 1D through H). However, there was no significant effect on serum insulin levels (Fig. 1I). Histological analysis via hematoxylin and eosin (HE) staining demonstrated that both BBR and MET administration alleviated hepatic lipid accumulation associated with diabetes (Fig. 1J). These findings are consistent with previous studies, which collectively suggest that both BBR and MET can improve glucose-lipid metabolism disorders in metabolic disorders mice.

### Relative quantitative sequencing-based observation of the effects of MET and BBR on the gut microbiota of metabolic disorders mice

**Fig 2:**
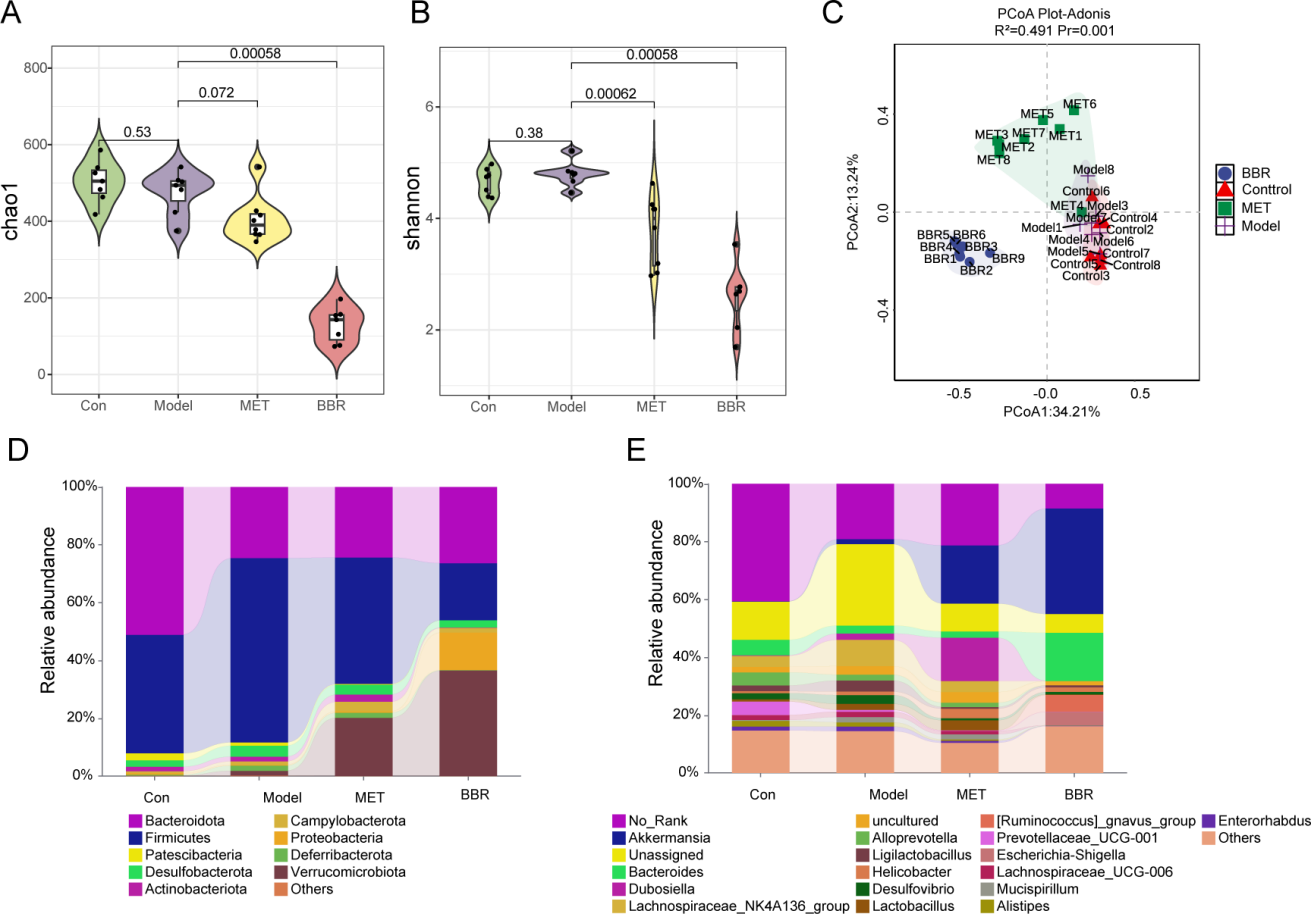
The gut microbiota diversity and overall structure regulated by BBR and MET treatment in relative quantitative analysis. (**A**) Chao1 index. (**B**) Shannon index. (**C**) Principal coordinates analysis (PCoA). (**D and E**) The taxonomic profiles of gut microbiota at the phylum (**D**) and genus (**E**) levels. “No rank” indicates that there is no clear classification information or classification name at a certain taxonomic level. “Unassigned” refers to bacteria that have not yet been cultured and identified.

Community richness was assessed using the Chao1 index, and diversity was evaluated using the Shannon index. Species richness and diversity in the high-fat diet group were reduced. Compared to the model group, MET supplementation did not significantly alter community richness although it significantly decreased diversity. In contrast, both richness and diversity of the gut microbiota were reduced after BBR supplementation, highlighting the antibacterial properties of BBR (Fig. 2A and B). The PCoA (Principal Coordinates Analysis) ordination coupled with Adonis permutation tests revealed significant effects of distinct experimental treatments on microbial community structure under relative quantitative sequencing (*R*² = 0.491, *P* = 0.001). Visualizations demonstrated clear spatial separation among sample points from the BBR-treated group, blank control group, and MET model group, with intra-group samples forming independent clusters. Notably, the 95% confidence ellipses exhibited no substantial overlap, corroborating the high inter-group heterogeneity identified by Adonis. Of particular interest, the grouping variable explained 49.1% of community variation (*R*^2^ > 0.3), indicating that experimental interventions (MET, BBR administration) or model establishment exerted robust remodeling effects on gut microbiota architecture (Fig. 2C). At the phylum and genus levels, microbiota composition was analyzed based on ASV abundance. The three predominant phyla were Bacteroidota, Firmicutes, and Verrucomicrobiota, and the relative abundance of Verrucomicrobiota was significantly increased after BBR and MET treatments (Fig. 2D). However, in the BBR and MET groups, there was an increase in the abundance of *Akkermansia* at the genus level. Additionally, *Bacteroides* abundance was notably higher in the BBR group (Fig. 2E).

**Fig 3:**
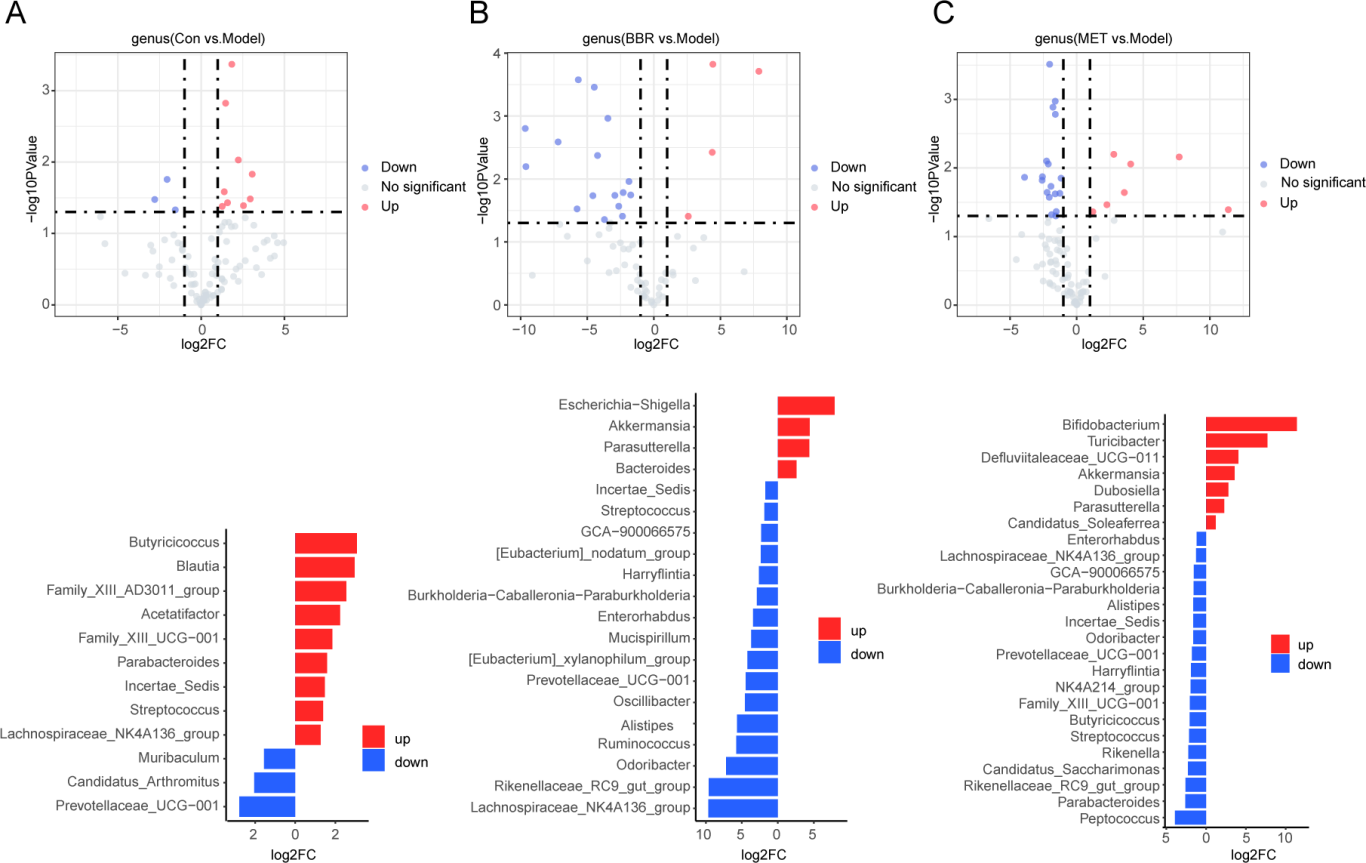
The core species of gut microbiota regulated by BBR and MET in relative quantitative analysis. (**A**) Upregulated and downregulated species in normal groups compared to the model group. (**B**) Volcano map and two-way bar chart showing the core species altered by BBR. (**C**) Volcano map and two-way bar chart showing the core species altered by MET.

Volcano plots were employed to compare species exhibiting significant changes in abundance across different groups. Following high-fat diet treatment, the abundance of beneficial bacteria, such as *Lachnospiraceae_NK4A136_group*, *Streptococcus*, *Parabacteroides*, *Butyricococcus*, *Blautia*, and other bacteria negatively correlated with obesity, non-alcoholic fatty liver disease (NAFLD), diabetes, and related diseases, showed a notable decrease (Fig. 3A). Upon administration of BBR, the abundance of gut commensal bacteria *Parabacteroides*, which produce secondary bile acids, and *Butyricococcus* and *Blautia*, which produce short-chain fatty acids, was significantly reduced. Conversely, the abundance of species such as *Rikenellaceae_RC9_gut_group*, *Lachnospiraceae_NK4A136_group*, *Odoribacter*, *Mucispirillum*, *Burkholderia-Caballeronia-Paraburkholderia*, *Streptococcus*, and others was notably downregulated (Fig. 3B). MET administration significantly upregulated the abundance of bacteria with potential roles in regulating bile acid and cholesterol metabolism, including *Lachnospiraceae_NK4A136_group*, *Streptococcus*, *Parabacteroides*, *Butyricicoccus,* and *blautia*. However, this treatment also resulted in a marked reduction in the abundance of several short-chain fatty acid (SCFA)-producing bacteria, such as *Lachnospiraceae_NK4A136_group*, *Alistipes*, *NK4A214_group*, *Butyricococcus*, *Streptococcus*, *Parabacteroides*, among others (Fig. 3C).

### Absolute quantitative sequencing-based observation of the effects of MET and BBR on the gut microbiota of metabolic disorder mice

The Chao1 index is utilized for assessing community richness, while diversity is evaluated using the Shannon index. Based on the Chao1 and Shannon indices, the species richness and diversity of the intestinal microbiota in metabolic disorder mice were found to be reduced. Compared to the model group, MET supplementation resulted in a decrease in community richness, though the difference was nozt statistically significant, while diversity was significantly reduced. In contrast, BBR supplementation led to a marked decrease in both the abundance and diversity of the gut microbiota (). Principal Coordinates Analysis (PCoA) combined with Adonis permutation tests was employed to evaluate the significant effects of distinct experimental treatments on microbial community structure under absolute quantitative sequencing (*R*² = 0.435, *P* = 0.001). Visualization revealed clear spatial separation among sample points from the BBR-treated group, blank control group, and MET model group, with intra-group samples forming distinct clusters. Furthermore, the 95% confidence ellipses exhibited no significant overlap, further validating the high inter-group heterogeneity identified by Adonis. The grouping variable explained 43.5% of the community variation (*R*² > 0.3), demonstrating that experimental interventions (MET induction, BBR administration) or model establishment exerted significant restructuring effects on gut microbiota architecture ().zFig. 4A and BFig. 4C

**Fig 4:**
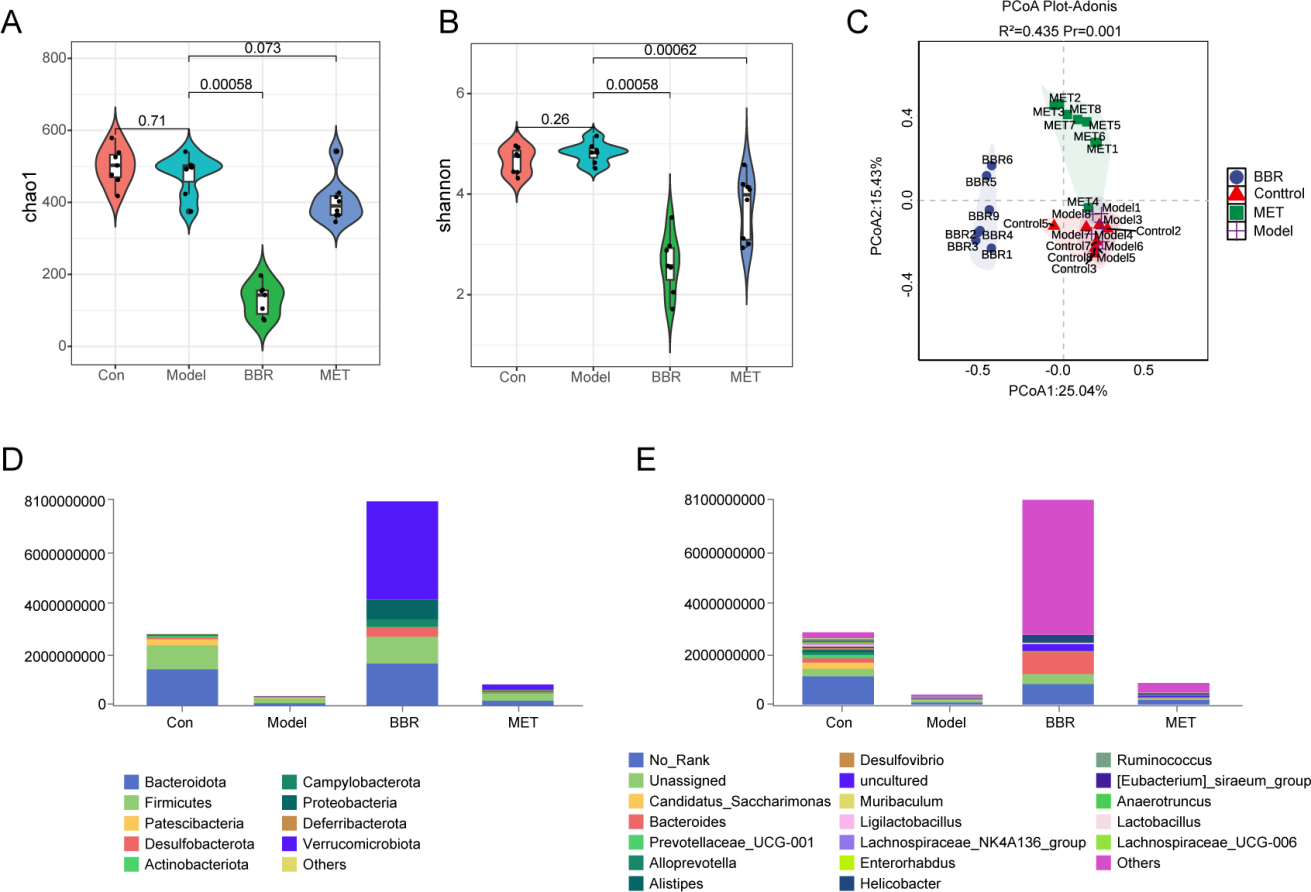
The gut microbiota diversity and overall structure regulated by BBR and MET treatment in absolute quantitative analysis. (**A**) Chao1 index. (**B**) Shannon index. (**C**) Principal coordinates analysis (PCoA). (**D and E**) The taxonomic profiles of gut microbiota at the phylum (**D**) and genus (**E**) levels. “No rank” indicates that there is no clear classification information or classification name at a certain taxonomic level. “Unassigned” refers to bacteria that have not yet been cultured and identified.

At the phylum level, the microbiota composition in each group was primarily dominated by the phyla Bacteroidota and Firmicutes although their absolute abundances varied across the groups. Notably, the total species count in both the model and MET groups was considerably lower compared to the control group. In contrast, the BBR group exhibited a significant increase in total species, particularly in the abundance of Verrucomicrobiota, Desulfobacterota, and Proteobacteria (Fig. 4D). At the genus level, the distribution of species mirrored the trends observed at the phylum level. Specifically, a high-sugar and high-fat diet leads to a reduction in the absolute quantitative abundance of gut microbiota, while BBR and MET treatments notably enhanced the abundance of Bacteroides (Fig. 4E).

**Fig 5:**
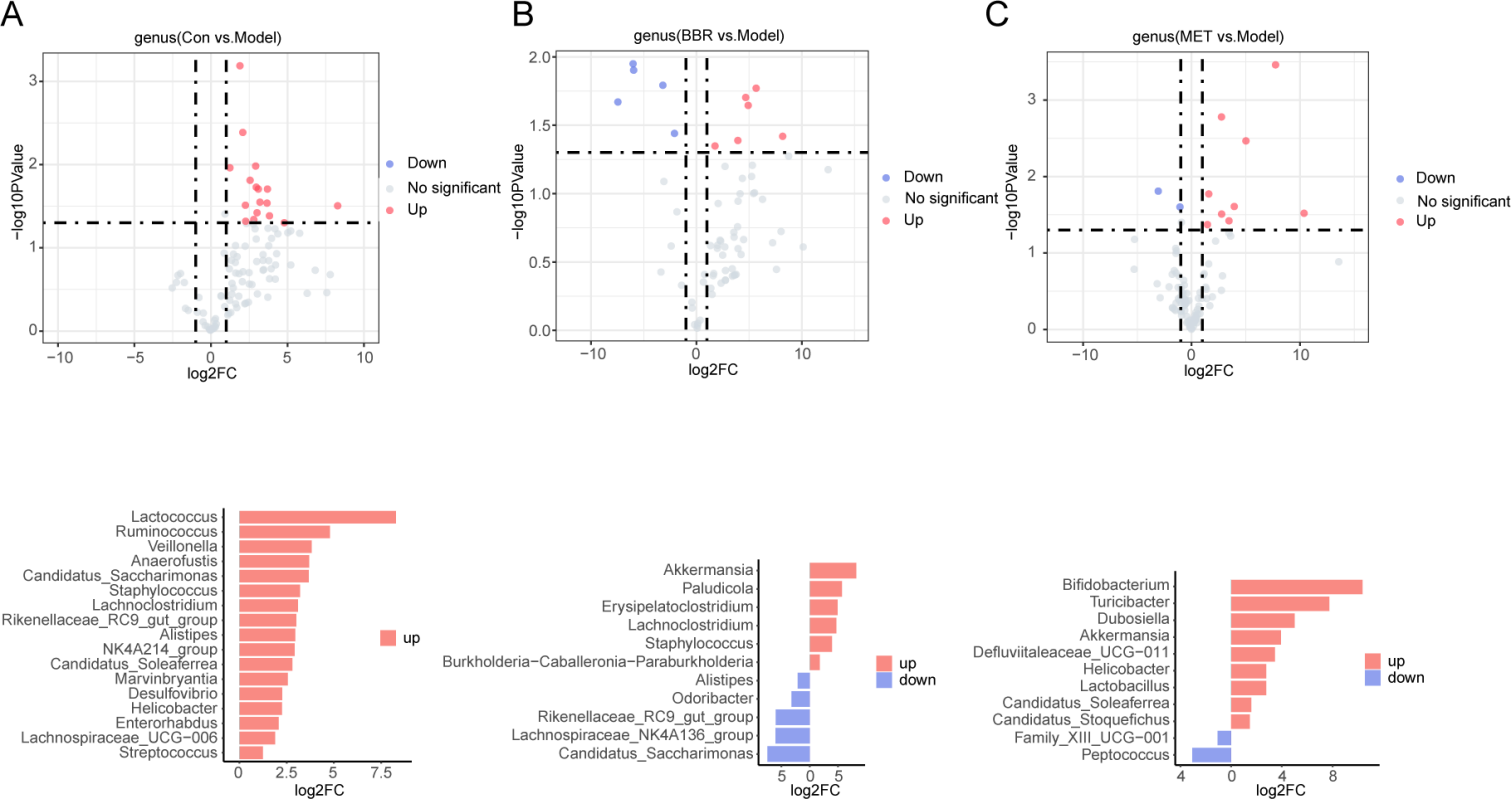
The core species of gut microbiota regulated by BBR and MET in absolute quantitative analysis. (**A**) Upregulated and downregulated species in normal groups compared to DSS group. (**B**) Volcano map and two-way bar chart showing the core species altered by BBR. (C) Volcano map and two-way bar chart showing the core species altered by MET.

Volcano plots and histograms were used to compare species with significant changes in abundance between groups. The abundance of *Lactococcus*, *Ruminococcus*, *Candidatus_Saccharimonas*, *Veillonella*, *Staphylococcus*, *Lachnoclostridium*, *Alistipes*, *Rikenellaceae_RC9_gut_group*, *NK4A214_group*, *Candidatus_Soleaferrea*, *Candidatus_Saccharimonas*, *Marvinbryantia*, *Helicobacter*, *Desulfovibrio*, *Lachnospiraceae_UCG-006,* and *Streptococcus* decreased significantly after high sugar and high fat feed treatment (Fig. 5A). The abundance of *Alistipes* and *Candidatus_Saccharimonas* was consistent with the model group and increased following BBR administration. Notably, the abundance of *Staphylococcus* was significantly elevated. Additionally, the abundance of several butyrate-producing bacteria, including *Akkermansia*, *Lachnoclostridium*, and *Burkholderia_Caballeronia_Paraburkholderia*, among others, showed a significant increase (Fig. 5B). MET supplementation significantly increased the reduction in *Helicobacter* abundance induced by a high-sugar, high-fat diet. In addition, MET significantly upregulated the abundance of SCFA-producing and mucin-degrading bacteria, such as *Bifidobacterium*, *Dubosiella*, *Akkermansia*, *Lactobacillus*, and downregulated the abundance of *Peptococcus* and *Family_XII_UCG-001*, which may be related to the lipid-lowering mechanism of MET (Fig. 5C).

### Difference between absolute quantification and relative quantification

Both relative quantitative sequencing and absolute quantitative sequencing showed no significant difference in the chao1 index of the community after supplementing MET, while the Shannon index significantly decreased. Consistent with previous findings, both Chao1 index and Shannon index decreased after BBR administration, highlighting the profound impact of BBR in altering individual-specific microbial fingerprints (33, 34). Principal Coordinates Analysis (PCoA) coupled with Adonis permutation tests demonstrated that the grouping variable explained over 40% of community variation (*R*² > 0.3, *P* < 0.001) in both absolute and relative quantitative sequencing data sets. This robust statistical evidence indicates that experimental interventions (MET induction, BBR administration) or model establishment exerted significant restructuring effects on gut microbiota architecture (Fig. 2A through C and 4A through C). These findings suggest that both relative and absolute quantitative sequencing methods demonstrate consistency in assessing the similarity or dissimilarity of community compositions across samples.

However, a significant difference in species composition was observed between the two measurement methods. In absolute quantitative sequencing, the species abundance in the normal group was substantially higher than in the model group (Fig. 2D and 4D). Post-administration of BBR, both the total number of species and the abundance of Verrucomicrobiota increased relative to the model group. After BBR administration, there was a marked increase in species abundance, particularly in Verrucomicrobiota, Desulfobacterota, and Proteobacteria at the phylum level, as well as *Bacteroides* at the genus level (Fig. 2E and 4E).

Although the differential bacterial abundances between groups, as measured by relative quantification, do not fully align with those observed through absolute quantification, some intestinal bacteria exhibit consistent regulatory trends across both methods. For instance, the abundance of Streptococcus was similarly downregulated in the model groups under both quantification modes (Fig. 3A and 5A). Following BBR administration, relative quantification revealed a significant increase in the abundance of only four bacterial species, whereas absolute quantification indicated a significant increase in six species. Notably, the abundance of *Akkermansia* was significantly upregulated in both quantification modes. Relative quantitative sequencing revealed a significant downregulation of BBR in 16 bacterial species, whereas absolute quantitative analysis identified a downregulation in only four species. Notably, the abundances of *Alistipes*, *Odoribacter*, *Rikenellaceae_RC9_gut group*, and *Lachnospiraceae_NK4A136_group* were consistently downregulated in both sequencing modes. Interestingly, while the relative quantitative sequencing indicated a decrease in the abundance of *Burkholderia-Caballeronia-Paraburkholderia*, absolute quantitative sequencing showed a significant increase in its abundance (Fig. 3B and 5B). This discrepancy highlights the limitation of relative abundance metrics, which do not accurately reflect the true microbial composition or the actual differences between samples across groups. Such variations may lead to biased interpretations and potentially erroneous conclusions.

The results of relative quantitative sequencing revealed that 7 bacterial species were upregulated, while 18 species were downregulated following MET administration. In contrast, absolute quantitative sequencing identified nine species that were upregulated and only two that were downregulated after MET treatment. Co-upregulated bacteria across both sequencing methods included *Bifidobacterium*, *Turicibacter*, *Dubosiella*, *Defluvitaleaceae_UCG-011*, *Akkermansia*, and *Candidatus_Soleaferrea*. Meanwhile, both sequencing approaches consistently showed that *Peptococcus* and *Family_XII_UCG-001* were downregulated (Fig. 3C and 5C). Additionally, we observed proliferative effects of MET and BBR on *Akkermansia* in both relative and absolute quantitative analyses. Furthermore, the absolute quantitative sequencing results demonstrated that BBR downregulated a significantly greater number of bacterial species compared to MET, whereas MET upregulated a higher number of species than BBR (Fig. 3B, C, 5B and C). This discrepancy helps elucidate the antibacterial properties of BBR and the growth-promoting effects of MET on specific bacterial populations, which is more in line with the reality.

### Conclusion

Our findings align with previous studies, demonstrating that both BBR and MET can effectively mitigate the symptoms of metabolic disorder. However, we observed significant differences in beta diversity and microbiota composition between relative and absolute quantitative sequencing methods. Furthermore, the comparison between the results of absolute and relative quantitative analyses reinforces the notion that relative quantitative sequencing is susceptible to misjudgments and false correlations. Overall, our study underscores the importance of absolute quantitative analysis in accurately representing true microbial counts in samples and evaluating the modulatory effects of drugs on the microbiome, which plays a vital role in microbiome research.

## DISCUSSION

Gut microbiota is increasingly recognized as a promising target for various diseases; however, current analyses of gut flora largely rely on relative quantitative methods. In our study, we first confirmed the beneficial effects of BBR and MET on metabolic disorders. Subsequently, we compared the regulatory effects of BBR and MET on gut microbiota using both relative and absolute quantitative methods, as well as comparing the sequencing results. Our findings indicate that while relative quantitative analysis can reveal alterations in bacterial communities, it fails to accurately reflect changes in the abundance of intestinal bacteria and may even display a completely opposite trend compared to absolute quantitative sequencing. This limitation overlooks the overall shifts in microbial communities, potentially resulting in the loss of critical information and leading to erroneous conclusions. Conversely, absolute quantitative methods provide a more precise representation of the actual changes in the bacterial community within the sample. Therefore, absolute quantitative analysis is essential in the study of bacterial communities.

The gut microbiota constitutes an extraordinarily complex ecosystem, and the aggregate of all gut microbiota genes (i.e., the microbiome) within an individual represents a genetic reservoir with an order of magnitude greater number of genes than that found in the human genome (20). Dysbiosis of the gut microbiota can contribute to various prevalent metabolic disorders, including obesity, T2D, non-alcoholic liver disease, metabolic heart disease, and malnutrition (35, 36). Consequently, sequencing methods for gut microbiota are vital for studying these microorganisms and their related functions. Currently, the majority of quantitative studies on gut microbiota employ relative quantitative methods (10).

Our results demonstrate that both relative and absolute quantitative sequencing methods exhibited consistency in assessing the similarity of community composition between samples. However, significant differences in species composition were observed between the two approaches. In comparison to the relative quantitative sequencing results, the absolute quantitative sequencing revealed that BBR had a more pronounced inhibitory effect on the proliferation of specific species (37), while MET showed a greater growth-promoting effect on certain colonies, such as *Bifidobacterium* and *Akkermansia,* which is consistent with previous research findings (38, 39). This finding is more conducive to understanding the intrinsic properties of the drugs. Meanwhile, our study identified a significant upregulation of *Akkermansia* in both absolute and relative quantitative sequencing, suggesting that *Akkermansia* plays a pivotal role for MET and BBR to alleviate metabolic disorders by regulating gut microbiota. These findings are in line with previous studies that have demonstrated that the total protein of *Akkermansia* can alleviate T2DM symptoms by activating the G protein-coupled receptor (GPCR) signaling pathway, thereby enhancing the synthesis and secretion of GLP-1 (40, 41). The above results indicate that, although relative quantitative sequencing-derived compositional data may exhibit spurious correlations during subsetting or aggregation, along with heightened sensitivity to sparsity and other analytical limitations, it remains capable of yielding biologically meaningful insights when appropriately contextualized (42). Furthermore, due to its cost-effectiveness and compatibility with high-throughput sequencing and the emergence of tools that account for the compositional nature of the data (e.g., SPieCeasi, SparCC network analysis) now enables robust interrogation of microbial community dynamics (43, 44). Consequently, relative quantification remains the core method for analyzing changes in microbial community structure, especially in large-scale longitudinal studies and multi-omics integration workflows (45).

In the absolute quantitative sequencing, the abundance of species in the normal group was significantly higher than that in the model group. This suggests that a prolonged high-sugar, high-fat diet may lead to a reduction in the overall abundance of the gut microbiota. Notably, most previous studies have focused primarily on changes in the abundance of beneficial or conditionally pathogenic bacteria in the intestines of patients with T2D, rather than examining the absolute abundance of enterobacteria in T2D patients themselves (46).

More importantly, our analysis revealed a notable discrepancy between relative and absolute quantitative sequencing results. Specifically, while the abundance of *Burkholderia-Caballeronia-Paraburkholderia* was downregulated in the relative quantitative sequencing data following BBR administration, it was found to be upregulated in the absolute quantitative sequencing results. This contrast highlights that relative and absolute quantitative sequencing approaches can lead to entirely opposing conclusions. Consequently, it is essential to carefully select appropriate analytical methods when studying the microbiome and investigating the regulatory effects of drugs on microbial communities.

## Data Availability

The raw sequencing data from the study can be found at the following location: the National Center for Biotechnology Information (NCBI) BioProject database, under the accession number PRJNA1248599.

## References

[1] Zou L-E, Yang Y-N, Zhan J, Cheng J, Fu Y, Cao Y, Yan X, Wang Y, Wu C. 2024. Gut microbiota-based discovery of Houttuyniae Herba as a novel prebiotic of Bacteroides thetaiotaomicron with anti-colitis activity. Biomed Pharmacother 172:116302. doi:10.1016/j.biopha.2024.116302 38387133

[2] de Vos WM, Tilg H, Van Hul M, Cani PD. 2022. Gut microbiome and health: mechanistic insights. Gut 71:1020–1032. doi:10.1136/gutjnl-2021-326789 35105664 PMC8995832

[3] Satinsky BM, Gifford SM, Crump BC, Moran MA. 2013. Use of internal standards for quantitative metatranscriptome and metagenome analysis. Methods Enzymol 531:237–250. doi:10.1016/B978-0-12-407863-5.00012-5 24060124

[4] Valles-Colomer M, Darzi Y, Vieira-Silva S, Falony G, Raes J, Joossens M. 2016. Meta-omics in inflammatory bowel disease research: applications, challenges, and guidelines. ECCOJC 10:735–746. doi:10.1093/ecco-jcc/jjw024 26802086

[5] Hu Q, Luo J, Cheng F, Wang P, Gong P, Lv X, Wang X, Yang M, Wei P. 2024. Spatial profiles of the bacterial microbiota throughout the gastrointestinal tract of dairy goats. Appl Microbiol Biotechnol 108:356. doi:10.1007/s00253-024-13200-8 38822843 PMC11144141

[6] Zhong S, Sun YQ, Huo JX, Xu WY, Yang YN, Yang JB, Wu WJ, Liu YX, Wu CM, Li YG. 2024. The gut microbiota-aromatic hydrocarbon receptor (AhR) axis mediates the anticolitic effect of polyphenol-rich extracts from Sanghuangporus. Imeta 3:e180. doi:10.1002/imt2.180 38882491 PMC11170970

[7] Zhao Y, Zhan J, Sun C, Zhu S, Zhai Y, Dai Y, Wang X, Gao X. 2024. Sishen Wan enhances intestinal barrier function via regulating endoplasmic reticulum stress to improve mice with diarrheal irritable bowel syndrome. Phytomedicine 129:155541. doi:10.1016/j.phymed.2024.155541 38579640

[8] Tkacz A, Hortala M, Poole PS. 2018. Absolute quantitation of microbiota abundance in environmental samples. Microbiome 6:110. doi:10.1186/s40168-018-0491-7 29921326 PMC6009823

[9] Stämmler F, Gläsner J, Hiergeist A, Holler E, Weber D, Oefner PJ, Gessner A, Spang R. 2016. Adjusting microbiome profiles for differences in microbial load by spike-in bacteria. Microbiome 4:28. doi:10.1186/s40168-016-0175-0 27329048 PMC4915049

[10] Vandeputte D, Kathagen G, D’hoe K, Vieira-Silva S, Valles-Colomer M, Sabino J, Wang J, Tito RY, De Commer L, Darzi Y, Vermeire S, Falony G, Raes J. 2017. Quantitative microbiome profiling links gut community variation to microbial load. Nature 551:507–511. doi:10.1038/nature24460 29143816

[11] Maghini DG, Dvorak M, Dahlen A, Roos M, Doyle B, Kuersten S, Bhatt AS. 2024. Quantifying bias introduced by sample collection in relative and absolute microbiome measurements. Nat Biotechnol 42:328–338. doi:10.1038/s41587-023-01754-3 37106038

[12] Zhan J, Cheng J, Chang W, Su Y, Yue X, Wu C. 2025. Absolute quantitative metagenomic analysis provides more accurate insights for the anti-colitis effect of berberine via modulation of gut microbiota. Biomolecules 15:400. doi:10.3390/biom15030400 40149936 PMC11940175

[13] Lou J, Yang L, Wang H, Wu L, Xu J. 2018. Assessing soil bacterial community and dynamics by integrated high-throughput absolute abundance quantification. PeerJ 6:e4514. doi:10.7717/peerj.4514 29576979 PMC5857175

[14] Morton JT, Sanders J, Quinn RA, McDonald D, Gonzalez A, Vázquez-Baeza Y, Navas-Molina JA, Song SJ, Metcalf JL, Hyde ER, Lladser M, Dorrestein PC, Knight R. 2017. Balance trees reveal microbial niche differentiation. mSystems 2:e00162-16. doi:10.1128/mSystems.00162-16 28144630 PMC5264246

[15] Gloor GB, Wu JR, Pawlowsky-Glahn V, Egozcue JJ. 2016. It’s all relative: analyzing microbiome data as compositions. Ann Epidemiol 26:322–329. doi:10.1016/j.annepidem.2016.03.003 27143475

[16] Harmsen HJM, Pouwels SD, Funke A, Bos NA, Dijkstra G. 2012. Crohn’s disease patients have more IgG-binding fecal bacteria than controls. Clin Vaccine Immunol 19:515–521. doi:10.1128/CVI.05517-11 22336288 PMC3318288

[17] Props R, Kerckhof F-M, Rubbens P, De Vrieze J, Hernandez Sanabria E, Waegeman W, Monsieurs P, Hammes F, Boon N. 2017. Absolute quantification of microbial taxon abundances. ISME J 11:584–587. doi:10.1038/ismej.2016.117 27612291 PMC5270559

[18] Tao Y, Zeng Y, Zeng R, Gou X, Zhou X, Zhang J, Nhamdriel T, Fan G. 2025. The total alkaloids of Berberidis Cortex alleviate type 2 diabetes mellitus by regulating gut microbiota, inflammation and liver gluconeogenesis. J Ethnopharmacol 337:118957. doi:10.1016/j.jep.2024.118957 39426578

[19] Yong Z, Ruiqi W, Yanan Y, Ning M, Zhi Z, Yinfeng T, Lin D, Yiying L, Weiying L, Chongming W, Xiaopo Z. 2022. Laurolitsine ameliorates type 2 diabetes by regulating the hepatic LKB1-AMPK pathway and gut microbiota. Phytomedicine 106:154423. doi:10.1016/j.phymed.2022.154423 36075181

[20] Fan Y, Pedersen O. 2021. Gut microbiota in human metabolic health and disease. Nat Rev Microbiol 19:55–71. doi:10.1038/s41579-020-0433-9 32887946

[21] Sonnenburg JL, Bäckhed F. 2016. Diet-microbiota interactions as moderators of human metabolism. Nature 535:56–64. doi:10.1038/nature18846 27383980 PMC5991619

[22] Chen K, Wang H, Yang X, Tang C, Hu G, Gao Z. 2024. Targeting gut microbiota as a therapeutic target in T2DM: a review of multi-target interactions of probiotics, prebiotics, postbiotics, and synbiotics with the intestinal barrier. Pharmacol Res 210:107483. doi:10.1016/j.phrs.2024.107483 39521027

[23] Li HY, Zhou DD, Gan RY, Huang SY, Zhao CN, Shang A, Xu XY, Li HB. 2021. Effects and mechanisms of probiotics, prebiotics, synbiotics, and postbiotics on metabolic diseases targeting gut microbiota: a narrative review. Nutrients 13:3211. doi:10.3390/nu13093211 34579087 PMC8470858

[24] Zhang W, Xu JH, Yu T, Chen QK. 2019. Effects of berberine and metformin on intestinal inflammation and gut microbiome composition in db/db mice. Biomed Pharmacother 118:109131. doi:10.1016/j.biopha.2019.109131 31545226

[25] Induri SNR, Kansara P, Thomas SC, Xu F, Saxena D, Li X. 2022. The gut microbiome, metformin, and aging. Annu Rev Pharmacol Toxicol 62:85–108. doi:10.1146/annurev-pharmtox-051920-093829 34449247

[26] He Q, Dong H, Guo Y, Gong M, Xia Q, Lu F, Wang D. 2022. Multi-target regulation of intestinal microbiota by berberine to improve type 2 diabetes mellitus. Front Endocrinol (Lausanne) 13:1074348. doi:10.3389/fendo.2022.1074348 36465656 PMC9715767

[27] Collins SL, Stine JG, Bisanz JE, Okafor CD, Patterson AD. 2023. Bile acids and the gut microbiota: metabolic interactions and impacts on disease. Nat Rev Microbiol 21:236–247. doi:10.1038/s41579-022-00805-x 36253479 PMC12536349

[28] Zhang Y, Gu Y, Ren H, Wang S, Zhong H, Zhao X, Ma J, Gu X, Xue Y, Huang S, et al.. 2020. Gut microbiome-related effects of berberine and probiotics on type 2 diabetes (the PREMOTE study). Nat Commun 11:5015. doi:10.1038/s41467-020-18414-8 33024120 PMC7538905

[29] Cheng M, Ren L, Jia X, Wang J, Cong B. 2024. Understanding the action mechanisms of metformin in the gastrointestinal tract. Front Pharmacol 15:1347047. doi:10.3389/fphar.2024.1347047 38617792 PMC11010946

[30] Bolyen E, Rideout JR, Dillon MR, Bokulich NA, Abnet CC, Al-Ghalith GA, Alexander H, Alm EJ, Arumugam M, Asnicar F, et al.. 2019. Reproducible, interactive, scalable and extensible microbiome data science using QIIME 2. Nat Biotechnol 37:852–857. doi:10.1038/s41587-019-0209-9 31341288 PMC7015180

[31] Callahan BJ, McMurdie PJ, Rosen MJ, Han AW, Johnson AJA, Holmes SP. 2016. DADA2: high-resolution sample inference from Illumina amplicon data. Nat Methods 13:581–583. doi:10.1038/nmeth.3869 27214047 PMC4927377

[32] Jiang SQ, Yu YN, Gao RW, Wang H, Zhang J, Li R, Long XH, Shen QR, Chen W, Cai F. 2019. High-throughput absolute quantification sequencing reveals the effect of different fertilizer applications on bacterial community in a tomato cultivated coastal saline soil. Sci Total Environ 687:601–609. doi:10.1016/j.scitotenv.2019.06.105 31220714

[33] Ren H, Shi Z, Yang F, Wang S, Yuan F, Li T, Li M, Zhu J, Li J, Wu K, Zhang Y, Ning G, Kristiansen K, Wang W, Gu Y, Zhong H. 2024. Deciphering unique and shared interactions between the human gut microbiota and oral antidiabetic drugs. Imeta 3:e179. doi:10.1002/imt2.179 38882498 PMC11170963

[34] Chen L, Wang D, Garmaeva S, Kurilshikov A, Vich Vila A, Gacesa R, Sinha T, Segal E, Weersma RK, Wijmenga C, Zhernakova A, Fu J. 2021. The long-term genetic stability and individual specificity of the human gut microbiome. Cell 184:2302–2315. doi:10.1016/j.cell.2021.03.024 33838112

[35] Donati Zeppa S, Gervasi M, Bartolacci A, Ferrini F, Patti A, Sestili P, Stocchi V, Agostini D. 2024. Targeting the gut microbiota for prevention and management of type 2 diabetes. Nutrients 16:3951. doi:10.3390/nu16223951 39599740 PMC11597803

[36] Dong C, Yang Y, Wang Y, Hu X, Wang Q, Gao F, Sun S, Liu Q, Li L, Liu J, Tang Y, Zhang S, Wu C, Zhu H. 2023. Gut microbiota combined with metabolites reveals unique features of acute myocardial infarction patients different from stable coronary artery disease. J Adv Res 46:101–112. doi:10.1016/j.jare.2022.06.008 35750287 PMC10105070

[37] Luan Q, Qiao R, Wu X, Shan J, Song C, Zhao X, Zhao Y. 2024. Plant‐derived Chinese herbal hydrogel microneedle patches for wound healing. Small 20:e2404850. doi:10.1002/smll.202404850 39073298

[38] Xie X, Li W, Xiong Z, Xu J, Liao T, Sun L, Xu H, Zhang M, Zhou J, Xiong W, Fu Z, Li Z, Han Q, Cui D, Anthony DC. 2025. Metformin reprograms tryptophan metabolism via gut microbiome-derived bile acid metabolites to ameliorate depression-Like behaviors in mice. Brain Behav Immun 123:442–455. doi:10.1016/j.bbi.2024.09.014 39303815

[39] Tawulie D, Jin L, Shang X, Li Y, Sun L, Xie H, Zhao J, Liao J, Zhu Z, Cui H, Wen W. 2023. Jiang-Tang-San-Huang pill alleviates type 2 diabetes mellitus through modulating the gut microbiota and bile acids metabolism. Phytomedicine 113:154733. doi:10.1016/j.phymed.2023.154733 36870307

[40] Niu H, Zhou M, Ji A, Zogona D, Wu T, Xu X. 2024. Molecular mechanism of pasteurized Akkermansia muciniphila in alleviating type 2 diabetes symptoms. J Agric Food Chem 72:13083–13098. doi:10.1021/acs.jafc.4c01188 38829529

[41] Li J, Yang G, Zhang Q, Liu Z, Jiang X, Xin Y. 2023. Function of Akkermansia muciniphila in type 2 diabetes and related diseases. Front Microbiol 14:1172400. doi:10.3389/fmicb.2023.1172400 37396381 PMC10310354

[42] Gloor GB, Macklaim JM, Pawlowsky-Glahn V, Egozcue JJ. 2017. Microbiome datasets are compositional: and this is not optional. Front Microbiol 8:2224. doi:10.3389/fmicb.2017.02224 29187837 PMC5695134

[43] Friedman J, Alm EJ. 2012. Inferring correlation networks from genomic survey data. PLoS Comput Biol 8:e1002687. doi:10.1371/journal.pcbi.1002687 23028285 PMC3447976

[44] Kurtz ZD, Müller CL, Miraldi ER, Littman DR, Blaser MJ, Bonneau RA. 2015. Sparse and compositionally robust inference of microbial ecological networks. PLoS Comput Biol 11:e1004226. doi:10.1371/journal.pcbi.1004226 25950956 PMC4423992

[45] Thorsen J, Brejnrod A, Mortensen M, Rasmussen MA, Stokholm J, Al-Soud WA, Sørensen S, Bisgaard H, Waage J. 2016. Large-scale benchmarking reveals false discoveries and count transformation sensitivity in 16S rRNA gene amplicon data analysis methods used in microbiome studies. Microbiome 4:62. doi:10.1186/s40168-016-0208-8 27884206 PMC5123278

[46] Liu N, Yan X, Lv B, Wu Y, Hu X, Zheng C, Tao S, Deng R, Dou J, Zeng B, Jiang G. 2024. A study on the association between gut microbiota, inflammation, and type 2 diabetes. Appl Microbiol Biotechnol 108:213. doi:10.1007/s00253-024-13041-5 38358546 PMC10869376

